# Defect visualization of Cu(InGa)(SeS)_2_ thin films using DLTS measurement

**DOI:** 10.1038/srep30554

**Published:** 2016-08-01

**Authors:** Sung Heo, JaeGwan Chung, Hyung-Ik Lee, Junho Lee, Jong-Bong Park, Eunae Cho, KiHong Kim, Seong Heon Kim, Gyeong Su Park, Dongho Lee, Jaehan Lee, Junggyu Nam, JungYup Yang, Dongwha Lee, Hoon Young Cho, Hee Jae Kang, Pyung-Ho Choi, Byoung-Deog Choi

**Affiliations:** 1 Analytical Engineering Group, Samsung Advanced Institute of Technology, 130, Samsung-ro, Yeongtong-gu, Suwon-si, Gyeonggi-do, 443-803, Korea; 2 College of Information and Communication Engineering, Sungkyunkwan University, Cheoncheon-dong 300, Jangan-gu, Suwon 440-746, Korea; 3 PV Development Team, Energy Solution Business Division, Samsung SDI, 467, Beonyeong-ro, Cheonan-si, Chungcheongnam-do 331-330, Korea; 4 Department of Physics, College of Science and Technology, Kunsan National University, Kunsan, 54150, Korea; 5 Department of Physics, Dongguk University, 100-715, Korea; 6 Department of Physics, Chungbuk National University, Cheongju, 28644, Korea

## Abstract

Defect depth profiles of Cu (In_1−x_,Ga_x_)(Se_1−y_S_y_)_2_ (CIGSS) were measured as functions of pulse width and voltage via deep-level transient spectroscopy (DLTS). Four defects were observed, i.e., electron traps of ~0.2 eV at 140 K (E1 trap) and 0.47 eV at 300 K (E2 trap) and hole traps of ~0.1 eV at 100 K (H1 trap) and ~0.4 eV at 250 K (H2 trap). The open circuit voltage (V_OC_) deteriorated when the trap densities of E2 were increased. The energy band diagrams of CIGSS were also obtained using Auger electron spectroscopy (AES), X-ray photoelectron spectroscopy (XPS), and DLTS data. These results showed that the valence band was lowered at higher S content. In addition, it was found that the E2 defect influenced the V_OC_ and could be interpreted as an extended defect. Defect depth profile images provided clear insight into the identification of defect state and density as a function of depth around the space charge region.

Recently, there have been considerable advancements in performance and cost-effectiveness of thin-film solar cells composed of copper indium gallium diselenide (CIGS) cells. Commercialized versions of these cells exhibit power conversion efficiencies close to 14%^1^. Moreover, the highest efficiencies obtained for CIGS solar cells on glass substrates or polymer films have exceeded 20%. Furthermore, the efficiency of CIGS solar cells is still being increased by pioneering research groups and companies^2^,^3^,^4^.

However, despite the great progress in efficiency and optimization of the manufacture of CIGSS solar devices, material properties and device physics have generally been improved through the use of repetition and empirical experiments. The properties of the defects created in CIGSS during growth and their influence on device performance are still only partially understood^5^,^6^. Studies investigating defects in CIGSS have been conducted by several research groups in attempt to determine the relationship between device efficiency and defect type/energy. Attempts to determine the influence of interface state on open circuit voltage (V_OC_) have been conducted via low-temperature capacitance measurements, external quantum efficiency (EQE) measurements, and other defect-recognition measurement techniques. The effects of Ga/(Ga + In) ratio (GGI) and/or S/(S + Se) ratio (SSSe) have been studied, and a correlation between presence of defects and decrease in V_OC_ has been reported. When the GGI is higher than 0.3, additional Ga influences not only the band gap, but also the transport mechanism and the defects in the absorber material^7^,^8^. By increasing the donor formation energies, Ga favors the formation of acceptors^9^. The metastability of CIGSS solar cells under illumination or in dark storage is a representative characteristic related to defects in the surface and bulk of the absorber layer. For example, several hours of light exposure results in an increase in the photoconductivity of CIGSS films; these films return to the initial state upon annealing at 80 °C in the dark^10^. Light-soaking treatments can also be used to re-establish cell efficiency after thermal treatments^11^. However, this correlation is not clearly understood.

Researchers have investigated the properties of defects in the space charge region (SCR) by utilizing various capacitance measurement methods such as admittance spectroscopy (AS), transient photocapacitance spectroscopy (TPS), and capacitance-voltage (CV) measurements^12^,^13^,^14^,^15^,^16^,^17^,^18^. However, these methods can only explain qualitative characteristics and are unable to quantify the defect density or determine the type of defects present (i.e., acceptors or donors). Since the performance of thin-film photovoltaic cells (e.g., cadmium telluride (CdTe), hydrogenated amorphous silicon (a-Si:H), and CIGSS) is generally governed by the quality of the interface and the SCR in the absorber layer, certain research groups have employed deep-level transient spectroscopy (DLTS) in order to investigate the junction characteristics^19^. Since the DLTS results are obtained from various reverse biases and filling pulses in analysis of CIGS solar cells, it is better to interpret the DLTS data of the defect depth profile by combining the composition analysis method (i.e., AES, EDX) and bandgap analysis method (i.e., XPS).

In this study, we attempted to visualize the presence of defects in the absorber layer; particular attention was paid to defects around the depletion edge. We were able to measure the defect distribution shape, energy, and depth profile of the surface by analyzing the pulse width modulation, pulse height, and reverse bias at various temperature conditions via DLTS measurements. The results of this study contribute to the physical explanation of the role of Cu(In,Ga)(Se,S)_2_ defects and describe the way in which defects are distributed around the space charge region in the CIGSS absorber layer.

## Materials and Methods

The CIGSS absorber layer was formed on a Molybdenum (Mo)-coated glass substrate. For a monolithically integrated module, we scribed the Mo layer using a high-power laser (P1). The width between each P1-scribed area was optimized with regard to the desired current density and the thickness/quality of the transparent conducting oxide (TCO) layer. Precursor layers were deposited on the Mo back electrode layer via direct-current (DC) sputtering. The substrate size was 902 × 1602 mm^2^. CIGSS absorber layers with a thickness of approximately 1.5–1.7 μm were formed by reacting the Cu-Ga/In precursors with H_2_Se and H_2_S gases diluted with Ar gas. The Cu-Ga and In precursors were sequentially deposited on the Mo back electrode via DC sputtering. The final Cu/(Ga + In) ratio was optimized to 0.88~0.92, a Cu-poor condition. Selenization and sulfurization were performed using the followed procedures. The chamber was filled with H_2_Se/Ar gas and heated to 400 °C and 450 °C for the samples named PK and PJ, respectively. The first step (i.e., the selenization process) was then executed for 25–35 min.

The PK sample was placed in an inert gas condition during a temperature increase from 400 to 550 °C, and then sulfurization was performed after filling the chamber with sulfur gas. For the PJ sample, the chamber was filled with sulfur gas right after the selenization process. Therefore, the PJ sample was in a sulfur gas condition during the temperature increase from 450 °C to 550 °C. The second step was performed at 550–580 °C for 60–90 min in H_2_S/Ar gas conditions. The chamber was then allowed to cool to 300–350 °C in H_2_S/Ar conditions and then purged with Ar gas. The reactions were conducted in a specially designed reaction chamber that was able to endure the highly toxic H_2_Se gas environment. The buffer layer, used to reduce shunt paths and increase the interface quality, was grown on the absorber layer using a chemical bath deposition (CBD) process. The CBD solution used to deposit the Zn(OH, O, S)-based buffer layer on the CIGSS absorber layer was formed by dissolving ZnSO_4_, NH_4_, and thiourea (CH_4_N_2_S) in deionized (DI) water. The samples were pre-rinsed with DI water at room temperature. The CBD process was performed at 60 °C, and the resulting buffer layer thickness was between 3 and 5 nm. The series connections between the unit cells that were separated by the P1 scribing were formed by mechanical scribing (P2). The gap between the P1- and P2-scribed regions was minimized in order to reduce the dead area.

B-doped ZnO (BZO) was used as the TCO layer and was fabricated via low-pressure chemical vapor deposition (LPCVD). The thickness and sheet resistance of the TCO layer were 950 nm and 11–13 Ω/□, respectively. The final step for separating the unit cells was conducted using a mechanical scribing process (P3).

The prepared CIGSS samples were investigated using transmission electron microscopy (TEM), junction electron beam-induced current (JEBIC), AES, reflection electron energy loss spectra (R-EELS), and DLTS. For in-depth compositional analysis of the CIGSS layers, the sputter depth profile was measured using AES during argon ion beam sputtering at 3.0 keV. The sputter rate of the CIGSS samples was 0.7 nm/min. This approach is useful for quantitative analysis because it presents no matrix effects; however, standard samples are required for accurate quantitative analyses. We quantified the elemental contents of Cu, In, Ga, Se, and S using the inductively coupled plasma (ICP)-AES results. Depletion width and carrier diffusion length were characterized using JEBIC performed using an FEI Sirion scanning electron microscope (SEM) and a point electronic JEBIC system. An E-beam energy of 2 kV and a fixed current of 388 pA were used to excite a constant amount of carriers. The electrons generated using the SEM system bombarded the cleaved facet of the CIGSS device to generate electron-hole pairs (EHPs). The EHPs were collected at both electrodes as a current, and the carrier diffusion length was characterized using the SEM image and current depth profile. The experimental methods, such as current-voltage-temperature (IVT), capacitance-voltage (CV), drive-level capacitance profiling (DLCP), and external quantum efficiency (EQE), are described in the Supplementary Information.

In order to characterize the deep-level defects and visualize the CIGSS solar cell thin films, a DLTS experiment was performed with a PhysTech FT1030 DLTS system. The capacitance was measured using a modified 1 MHz Boonton 72B capacitance meter. Temperature scans were performed between 20 and 300 K, at a heating rate of 2 K/min. Samples were placed in the He-contact gas of a liquid helium cryostat. The pulse height, filling pulse width, and pulse period width were 0.2 V, 10 ms, and 10 ms, respectively. In order to capture the cross-section and defect density profile, we used different pulse widths ranging from 10^−3^ to 0.5 sec. The activation energy, capture cross-section, and concentration of traps were calculated using an Arrhenius plot.

## Results and Discussion

Table 1 shows the summary of the device specifications for the two samples (PJ and PK). The parameters were extracted from current-voltage (IV) and capacitance measurements, and the V_OC_ loss was calculated using the relation between Eg and V_OC_. The samples were classified according to difference in amounts of sulfur and gallium elements present, together with their cell efficiencies. The PJ sample exhibited a low efficiency of 16.09%, whereas the PK sample exhibited a higher efficiency of 16.62%. The short circuit current (J_SC_) and V_OC_ were 37.0 mA/cm^2^ and 0.650 V in the PJ sample and 37.03 mA/cm^2^ and 0.673 V in the PK sample, respectively.

**Figure 1:**
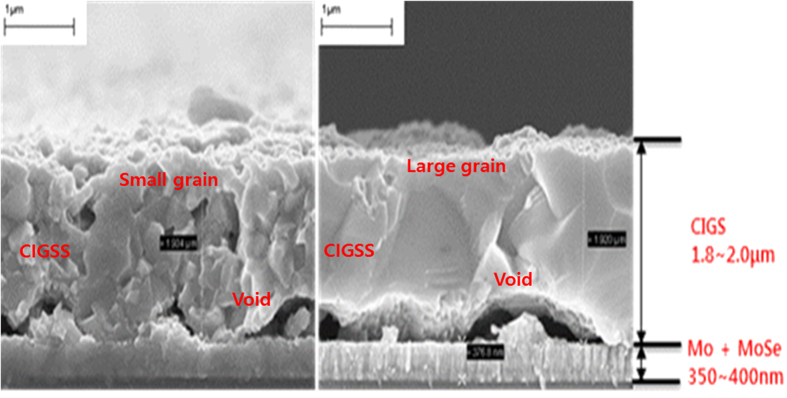
Cross-sectional SEM images of the (**a**) PJ and (**b**) PK CIGSS samples. Several voids were observed in the CIGSS layers and at the interface between the CIGSS and MoSe_2_ layers.

Figure 1 shows the cross-sectional SEM images of the (a) PJ and (b) PK CIGSS samples. Large voids were observed in all of the samples at the interface between the bottom CIGSS layer and the molybdenum (Mo) layer. These voids were typically formed due to the volume expansion (a near three-fold expansion) that occurs during the selenization process. The PK sample contained large grains in most regions, especially in the bottom CIGSS layer, compared with the PJ sample. Thus, the large grain size in the PK sample was due to selenization at a low temperature (400 °C). Kim *et al*.^20^ clearly described the mechanism of grain growth in low-temperature selenization. The Cu_9_Ga_4_ alloy remained at the bottom of the absorber layer because of partial selenization at low temperatures and then started to melt at approximately 490 °C in the inert gas condition. The Cu_9_Ga_4_ alloy was then present in the liquid phase, allowing grains to grow through the mechanism of Ostwald ripening via liquid-assisted grain growth, where component elements move very quickly through the liquid phase^20^,^21^. However, in the PJ sample, the Cu_9_Ga_4_ alloy did not remain in the absorber layer because of complete selenization at high temperatures; thus, there was no liquid phase. Therefore, there was no grain growth after the sulfurization process at high temperatures, i.e., 550 °C.

**Figure 2:**
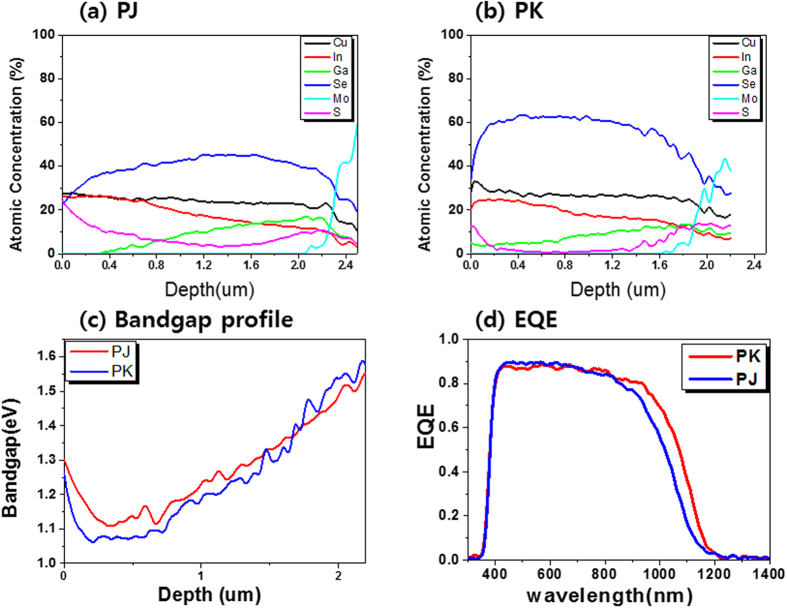
Auger depth profiles of (**a**) PJ and (**b**) PK samples. (**c**) The band gap profiling calculated from Auger depth profiling and (**d**) EQE spectra of samples PJ and PK.

Figure 2(a,b) show the quantified Auger depth profiles of the PJ and PK samples, respectively. The AES data clearly show a much higher peak for sulfur at the surface in the PJ sample than in the PK sample. In the PK sample, Ga appeared on the surface because it diffused outward to the surface via the liquid phase of Cu_9_Ga_4_ under partial selenization at low temperatures, leading to the presence of large grains^20^,^21^.

In order to determine the J_SC_ and V_OC_, which are the factors that affect device efficiency, we calculated the band gap profiles of the two samples based on the Auger sputter depth profiles.

Bar *et al*. reported^22^ an empirical band gap equation for the Cu(In_1−x_,Ga_x_)(Se_1−y_S_y_)_2_ system. The elemental composition was measured from the AES, and the results were used to produce Eq. (1).


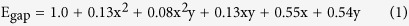


Figure 2(c) shows the band gap profile that was calculated using the Auger profiles of the PJ and PK samples. The AES band gap profile shows that the band gap increased toward the surface, gradually decreased in the direction of the bulk CIGSS (i.e., the space charge region), and then increased at the back of the CIGSS/Mo, producing a V-shaped double-graded band gap profile.

The minimum band gap of the PK sample was smaller than that of the PJ sample. As the minimum band gap decreased, the J_SC_ increased. This is in good agreement with the electrical properties shown in Table 1.

Figure 2(d) shows the external quantum efficiency (EQE) data. The EQE curves show that the PK sample absorbed a wider spectrum than the PJ sample due to its smaller band gap, which was calculated from the EQE. This result is in good agreement with the calculated AES band gap, as shown in Fig. 2(c).

**Figure 3:**
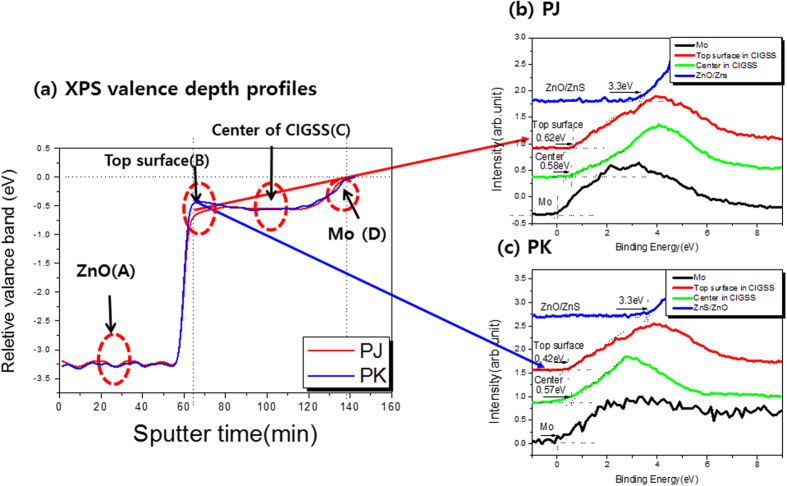
XPS valence depth profiles of (**a**) the PJ (red line in the figure) and the PK (blue line in the figure) samples. (**b**,**c**) XPS valence spectra in the ZnO (region A), the ZnO/CIGSS interface (i.e., the top surface of the CGSS layer, region B), the center of the CIGSS film (region C), and the Mo (region D) of the PJ and PK samples.

Figure 3(a) shows the XPS valance depth profile of the PJ (red line in the figure) and PK (blue line in the figure) samples. Figure 3(b,c) show the valence spectra in the ZnO (region A), the ZnO/CIGSS interface (i.e., the top surface of the CGSS layer, region B), the center of the CIGSS film (region C), and the Mo (region D) of the PJ and PK samples, respectively.

In order to determine the valence band maximum (VBM) offset of the ZnO/CIGSS/Mo structure, the valence band spectra were measured and shown to be reproducible within an uncertainty of 0.1 eV in a series of experiments, shown in Fig. 3(b,c). The VBM of the Fermi level was determined from the intersection of two straight lines fitting the valence band leading edge and the background.

In addition, the VBM value at the ZnO/CIGSS interface was lower for the PJ sample than for the PK sample (as indicated by the red dashed circle in Fig. 3(a)). This might have been caused by the presence of a larger amount of sulfur on the surface (Table 2). In general, the band gap in the CIGSS sample can be controlled by increasing the Ga composition in the back side and increasing the amount of sulfur (S) that is injected in the front side during the sulfurization process.

The band gap increased with increasing amount of Ga due to the increase in conduction band minimum (CBM), which determines the anti-bonding between the Ga 4s* and Se 4p* orbitals^23^,^24^.

An increased concentration of sulfur (S) not only increases the CBM, but also decreases the valence band maximum (VBM), since the VBM is associated with bonding between the Cu 3d, Se 4p, and S 3p orbitals^24^,^25^,^26^,^27^,^28^.

In addition, the valence band values at the CIGSS/Mo interface in the bottom region were similar in the two samples. There was no difference in valence band values (i.e., the contributions to the valence band of the Ga and S were similar in the two samples) because of the increases in Ga and S at the CIGSS/Mo interface.

Device performance is significantly influenced by both the band gap profile and the defect state. For this reason, it is important to understand the defect state in CIGSS cells. A DLTS experiment was carried out in order to analyze this defect state.

Table 1 shows the carrier concentration profiles that were obtained using capacitance-voltage (CV) measurements of the PJ and PK samples using 50 mV amplitude and 100 kHz frequency. Since the PK sample exhibited relatively lower carrier concentration profiles compared to the PJ sample, it contained a wider space charge region (SCR). The free carrier density and room temperature (RT) carrier density that were measured by drive-level capacitance profiling (DLCP) are listed in Table 1. The method employed for DLCP measurement is well described in the literature^29^. The number of free carriers was almost the same in both samples because of the use of identical precursor conditions. However, there was a significant difference in RT density between the two samples. The cause of the low RT density of the PK sample is not yet fully understood, but it is suspected that this is a grain boundary effect. In the DLCP measurement, interface defects were ignored because the given input signal only affected the bulk absorber area^29^. The difference between free carrier density and RT density could be due to the density of defects. However, DLCP measurements cannot be used to determine the origin of defects (acceptor or donor defects), although it is possible to measure the amounts of carriers and defects.

**Figure 4:**
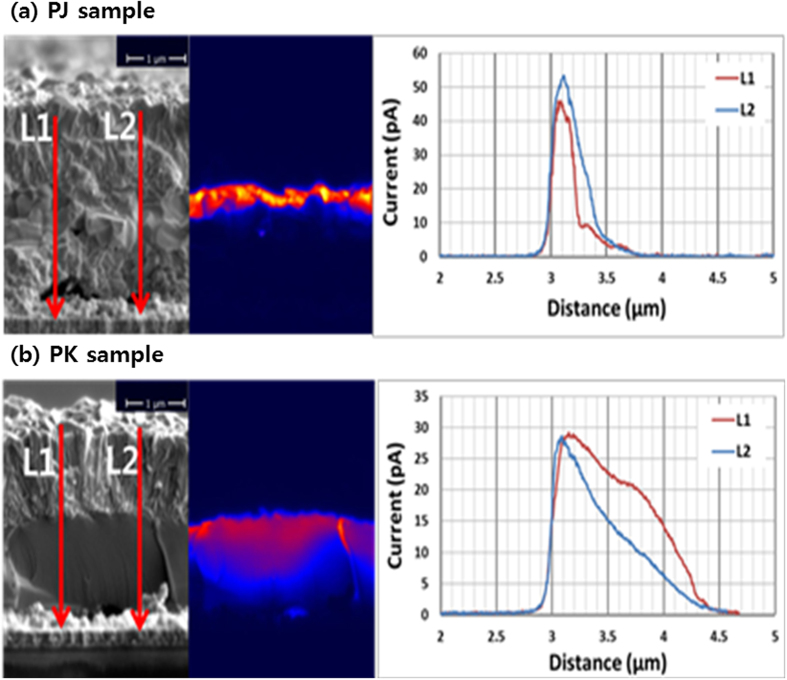
JEBIC images of the (**a**) PJ and (**b**) PK samples.

Figure 4(a,b) show the JEBIC data for samples PJ and PK, respectively, where the line profiles of both samples are described in a graph. JEBIC measurements were used to examine the carrier behaviors in the absorber area. JEBIC is a technique used to measure the currents that flow in a semiconductor when it is exposed to an electron beam. When an electron beam strikes a semiconductor, electron-hole pairs are created. If the electrons successfully diffuse into a space charge region, the electrons and holes will be collected by both electrodes, and a current will be created. Using the signal from a picoammeter as the imaging signal, a JEBIC image is formed on the display of an SEM. If the JEBIC signal of the SCR shows particularly high intensity, the carriers can be successfully separated and transferred to either side of the junction before recombination. However, in the quasi-neutral region (QNR), which exhibits a relatively low JEBIC intensity, the generated carriers are recombined through bulk defects via Shockley-Reed-Hall (SRH) paths. The PK and PJ samples presented significantly different characteristics in terms of JEBIC data. The PJ sample exhibited high intensity at the SCR region, and the intensity was drastically decreased at the QNR. Generated charges were collected up to 700 nm from the PJ sample. However, the PK sample exhibited relatively low intensity at the SCR, but its JEBIC profile was prolonged to the end of the absorber layer (1.5 μm). We assert that the long diffusion length of the generated carriers in the PK sample was caused by a large grain effect. Because of the presence of large grains, the generated carriers in the PK sample could conduct the carriers from the end of the absorber layer to the SCR, instead of through grain-to-grain hopping.

**Figure 5:**
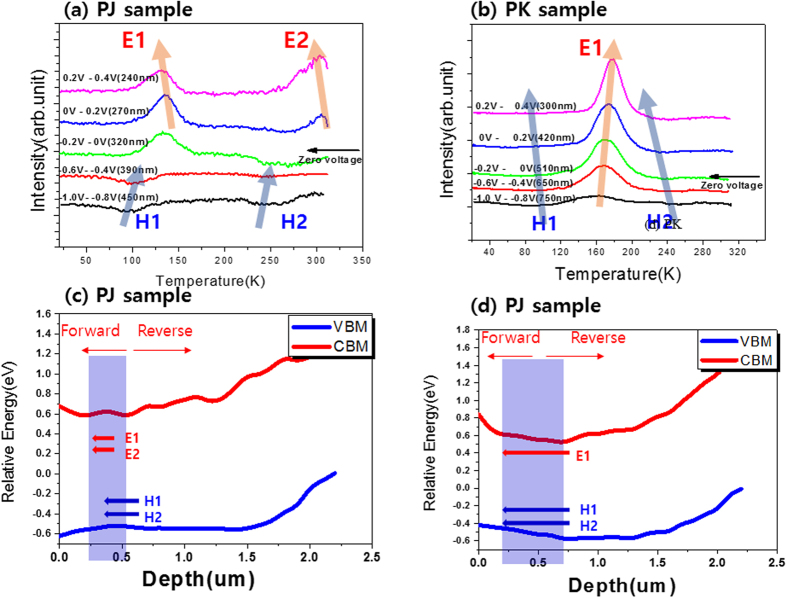
DLTS depth-profiling spectra with various bias voltages for the (**a**) PJ and (**b**) PK samples. Defect energy diagrams of the (**c**) PJ and (**d**) PK samples.

Figure 5(a,b) show the DLTS spectra of the PJ and PK samples, respectively, at various reverse and forward bias voltages at a pulse height fixed at 0.2 V. In the DLTS experiment, as the bias voltage changed, the depletion region (i.e., the SCR) also changed. Therefore, defects at various film depths can be investigated by changing the bias voltage, thereby allowing measurement of defect states from the surface to the bulk of the CIGSS layer. As shown in Fig. 5(a), in the reverse region of the PJ sample (i.e., the bottom of the depletion region in the CIGSS films), the H1 and H2 peaks were dominant; however, in the forward direction, the H1 and H2 peaks disappeared, and E1 and E2 peaks appeared. H1 defects are associated with acceptors and are thought to be related to copper vacancy (Vcu) defects, which are related to p-type defects in CIGSS thin films^7^,^30^,^31^.

In addition, we suggest that H2 defects are related to N2 defects, which have been defined as bulk acceptor defects in a previous paper^7^. M. Turcu *et al*.^7^ reported that the performance of solar cells is closely related to the concentration of bulk acceptor N2 defects (i.e., H2 defects in our experiment). In addition, E1 defects, which are interface defects in the ZnO/CIGSS region, are thought to be interface donor-like defects; these are referred to as N1 defects in the literature [7, 34]. Furthermore, the E2 peak, which also represents trap states at the interface, had a higher defect density in the forward direction compared to the peak representing E1 traps. As shown in Fig. 5(b), in the reverse region of the PK sample, H1 and H2 traps existed; however, at a forward bias, the H1 peak disappeared, and a new E1 peak appeared. As mentioned in the previous paragraph, the H1, H2, and E1 peaks are related to Vcu defects^30^, bulk acceptor defects^7^, and interface donor-like defects^31^, respectively.

According to a previous paper^32^, a p/n homojunction is formed within the CIGS film through Zn diffusion. In the XPS depth profile of the PJ sample, as shown in Supplementary Figure 3, Zn diffused into the CIGS thin film. Also, in the SCM data shown in Supplementary Figure 4, the contrast in the SCM image is the difference in CIGSS area in the PJ sample. The bright area of contrast indicates the p type species, and the dark areas represent the n type. Those results indicate that a homo-junction was formed within the CIGSS film; a p/n junction was formed because the upper depletion region in the CIGSS layer was n-type (caused by the E1 defect), while the lower depletion region in the CIGSS layer was p-type (caused by H1 and H2 defects).

From the results of the AES bandgap profile in Fig. 2(c), XPS valance band spectra in Fig. 3(a) and DLTS spectra in Fig. 5(a,b), the band diagram including defect energy levels shown in Fig. 5(c,d) in the PJ and PK samples, respectively.

The H1 and H2 peaks decreased in density and shifted to higher energy levels in the forward region. Additionally, the density of the E1 peak increased, and its energy shifted to a lower level in the forward region. In particular, the increasing density of the E2 peak rapidly increased at the depletion interface under a forward bias. Conversely, in the forward region of the PK sample (Fig. 5(b,d)), the densities of the H1 and H2 peaks decreased, and their activation energies shifted to lower values. Additionally, the E1 peak increased in density, and its activation energy was shifted to a higher energy. By analyzing the band gap and valence band obtained using the AES and XPS data, we can see that the conduction and valence bands tended to move downward together, toward the top surface in the depletion region of the CIGSS films in the PJ sample (Fig. 5 (c)). From the activation energy of the defect state obtained via DLTS in the forward region, we can see that the activation energy of electrons was decreased, while the activation energy of holes was increased. These results indicate that the defect energy level is independent of the values from the conduction band or valence band.

Figure 2(d) shows the EQE data obtained. We calculated the minimum energy gap of the absorber layer using the first derivative of the EQE curves. The band gap is described in Table 1. We infer that the PK sample can absorb a wider spectrum of wavelengths compared to the PJ sample because of its narrower band gap. In general, photovoltaic devices with wider band gaps exhibit higher V_OC_. However, the PK sample with a narrow band gap (1.13 eV) exhibited a higher V_OC_ compared to the PJ sample (1.181 eV). The PK sample contained a small amount of E1 defects in the reverse region, and the E1 defect intensity was higher when the DLTS scanning area was close to the interface between the absorber and buffer layer in the PK sample. Conversely, the PJ sample contained a relatively large amount of E2 defects compared to E1 defects in the forward region. As shown in Table 3, the activation energies for E1 and E2 were 0.18–0.23 eV and 0.47 eV, respectively. When we consider the E1 defects present in both samples, the energy and intensity of an E1 defect in the PK sample were almost the same as that in the PJ sample, and the capacitance signal of the E1 defect obtained using DLTS measurements was observed at low temperatures less than 200 K. Therefore, there was almost no performance loss caused by the E1 defects in either sample. However, the E2 defect signal was significant in the PJ sample, while there was only a very small signal for the PK sample. The E2 defect was located at a very deep energy level (~0.47 eV), which was close to the middle of the energy gap and was distinguished using high-temperature DLTS measurement (~300 K).

The Fermi-level pinning can be explained by the defect state in the metal and semiconductor^33^. The potential barriers can be obtained from C-V characteristics, as shown in Fig. 1, and were Ec-0.48 eV and Ec-0.15 eV for the PJ and PK samples, respectively. The Fermi level was aligned at the E2 level in the PJ sample and at the E1 level in the PK sample.

Also, for the PJ sample, the Fermi level was pinned to the energy level of E2 because of its comparatively higher density (relative to E1) in the forward region^28^. In the PJ sample, Fermi-level pinning was aligned at a deeper level (i.e., the activation energy of E2 is deeper than that of E1). The high-energy E2 defect located at the interface can act as a recombination source and can influence device performance through the degradation of V_OC_. Interface recombination occurred here due to the presence of the E2 defect with high energy/density. Therefore, the PJ sample exhibited a lower V_OC_, even though it exhibited a larger band gap (Table 1).

For a point defect, this can be expressed by the following equation^34^,^35^:


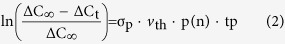


where ΔC_∞_ is the equilibrium capacitance value, ΔC_t_ is the capacitance at time t, σ_p_ is the trap capture cross-section, p(n) is the majority (minority) carrier concentration, tp is the filling pulse width, and ν_th_ is the thermal velocity.

In the case of a point defect, the majority capture cross-section should demonstrate a linear dependence on the logarithm of a combination of the capacitance terms as a function of pulse length.

However, in the case of an extended defect, the amplitude of the DLTS peak is proportional to the filling pulse width, as shown in the following equation^34^,^35^,^36^:


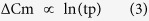


where ΔC_m_ denotes the amplitude of the DLTS signal.

The DLTS signal should be dependent on the logarithm of the filling pulse width when the carrier capture is allowed for extended defects.

**Figure 6:**
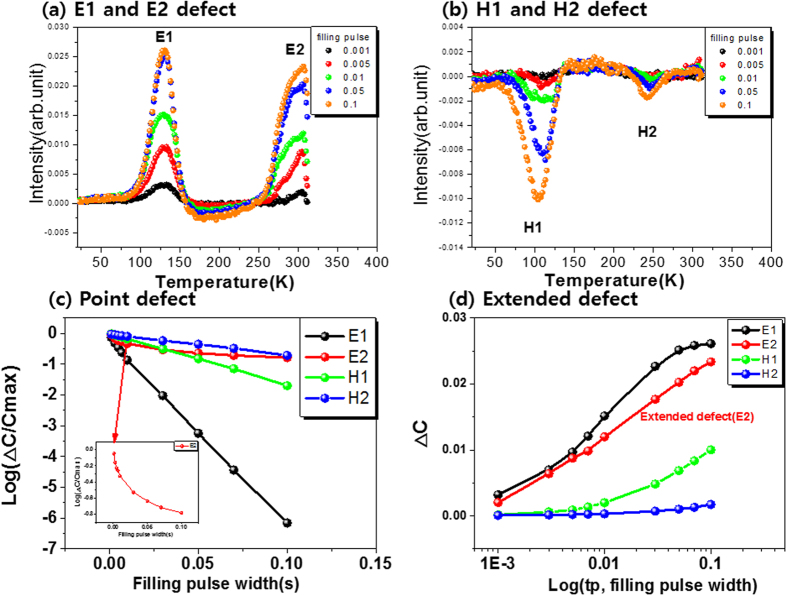
The DLTS spectra as a function of fill-pulse width of (**a**) the electron defect at E1 and E2 in the forward region (from 0.2 V to 0.4 V), (**b**) the hole defect at H1 and H2 in the reverse region (from −0.6 V to −0.4 V), and the (**c**) point defect and (**d**) extended defect for E1, E2, H1, and H2. Inset graph of (**c**) is an enlargement of the E2 peak, which is not linear. This result indicates that the E2 peak is an extended defect rather than a point defect.

Figure 6(a,b) show the DLTS spectra as a function of the fill-pulse width of the electron defects at E1 and E2 in the forward region (from 0.2 V to 0.4 V) and the hole defects at H1 and H2 in the reverse region (from −0.6 V to −0.4 V)

Figure 6 shows the (c) point defect and (d) extended defect for E1, E2, H1, and H2 peaks. The peaks of E1, H1, and H2 show excellent linearity when plotted in accordance with Eq. (2), as shown in Fig. 6(c). Based on these results, the E1, H1, and H2 defects can be defined as point defects. However, the E2 peak cannot be defined using Eq. (2) and exhibits very good linearity when defined in relation to Eq. (3), as shown in the insert of Fig. 6(d). The origin of E2 is unclear; however, our results indicate that the E2 defect could be an extended defect, such as a grain boundary-related defect, a dislocation, or a void. Oana Cojocaru-Miredin *et al*. reported^37^ rich Na compositions in the grain boundary, according to atom probe tomography measurement, which induced extended defects. L. E. Oikkonen *et al*. also reported^38^ that two (Na-Na)Cu dumbbells are slightly attracted to each other, which suggests that Na atoms could cluster together in polycrystalline material. This could occur at grain boundaries, resulting in extended defects consisting of several (Na-Na)Cu dumbbells with +1 charge. In addition, in the PJ sample, the grain in the top region of the CIGSS is considerably smaller than that in the PK sample; the grain boundary is thus thicker than that in the PK sample, as shown in the SEM image of Fig. 1. Conversely, the grain size in the PK sample is very large, and the grain boundary in the entire CIGS is very small. The grain boundary in the PJ sample is more than that of the PK sample. This suggests that the observed extended electron defects were impacted by the grain boundary area.

**Figure 7:**
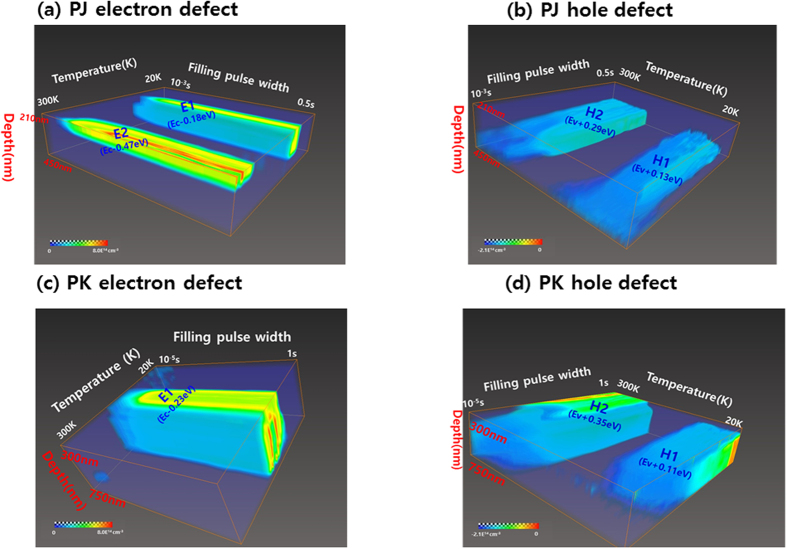
Defect imaging of electron and hole traps in the PJ ((**a**,**b**)) and PK ((**c**,**d**)) samples.

In order to capture images of defects, we used Avizo software, which is a 3D analysis software for scientific and industrial data, where the x-axis is the temperature related to the activation energy (from 20 K to 300 K), the y-axis is the filling pulse width related to the capture cross-section and defect density (from 10^−3^ s to 0.5 sec), the z-axis is the bias voltage related to the depletion depth (from −1.0 V to 0.6 V), and the color is defect density (from −2.1 × 10^14^ cm^−3^ to 8.0 × 10^14 ^cm^−3^). Figure 7(a,b) show defect images of electron traps and hole traps, respectively, in the PJ sample. A color change from blue to red indicates that the defect density increased. As the fill pulse was increased, the defect density increased. The lower region is in the reverse direction, and the upper region is in the forward direction of the top of the depletion region. Figure 7(a) depicts an electron trap and shows the appearance of E1 and E2 peaks; the density of the E2 peak increases more significantly in the forward direction than does the E1 peak. Figure 7(b) illustrates a hole trap, where the H1 and H2 peaks are detected in the reverse region and gradually disappear in the forward region.

**Figure 8:**
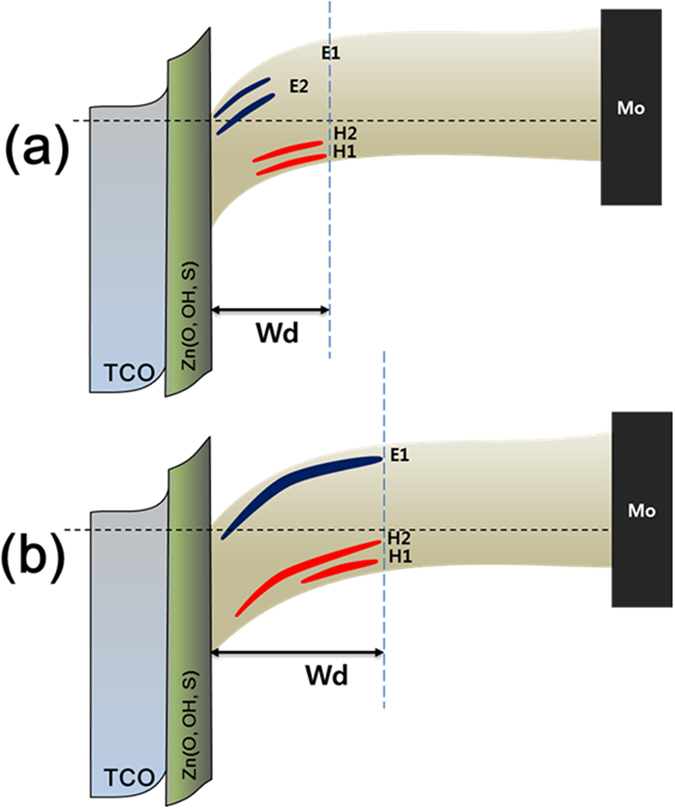
A replotted energy band diagram, obtained using the DLTS results for the (**a**) PJ and (**b**) PK samples.

In addition, Fig. 7(c,d) show images of an electron trap and hole trap for the PK sample, respectively. As shown in Fig. 7(c), the electron trap shows only the E1 peak, which exists in the full depletion region. Additionally, in Fig. 7(d), the H1 and H2 peaks are detected for a two-hole defect. In the forward region, the H1 defect gradually decreases, and the H2 defect appears to persist without disappearing. As shown in Fig. 7 for the PJ sample, the H1 and H2 defects exist in the back side of the depletion region and gradually disappear in the front side of the depletion region, while the E1 and E2 defects do not exist in the back side of the depletion region and gradually appear in the front side of the depletion region. However, in the PK sample, E1 and H2 peaks exist in the full depletion region. The H1 peak exists in the back side of the depletion region and gradually decreases in intensity in the front side of the depletion region. On the basis of these results, we replotted the energy band diagram, as shown in Fig. 8.

Figure 8 shows the replotted energy band diagram constructed using the DLTS results for the (a) PJ and (b) PK samples. The SCR region in the PK sample is larger than that in the PJ sample due to its lower carrier concentration. Additionally, the DLTS results show that the Fermi levels of the PJ and PK samples are aligned with the E2 and E1 peaks, respectively. We infer that the V_OC_ loss was caused by Fermi-level pinning at the E2 peak in the PJ sample.

## Conclusion

In order to investigate the optimization conditions of CIGSS solar cell devices, we quantitatively studied the band gap and valence band profiles and the defect levels using AES/XPS and DLTS, respectively.

The minimum band gap depth, observed by Auger depth profiling of the surfaces of the CIGSS layers, was determined to occur at 0.2 μm and 0.3 μm for the PJ and PK samples, respectively. The minimum band gap of the PK sample was smaller than that of the PJ sample, which was in good agreement with the EQE results.

On the surface of the CIGSS layer, the valence band maximum of the PJ sample was lower than that of the PK sample due to an increased concentration of sulfur in the PJ sample.

H1, H2, E1, and E2 peaks were observed in the PJ sample using DLTS. We note that the E2 peak intensity increased toward the ZnS/CIGSS interface, and that the Fermi level was pinned to the E2 peak. As a result, V_OC_ loss occurred. Also, the E2 defect can be interpreted as a grain boundary-related defect. In the PK sample, H1, H2, and E1 peaks were observed, but no E2 peak was observed. There was also an H2 peak at the ZnS/CIGSS interface; this defect might have been a bulk acceptor defect. We conducted defect depth-profile imaging by constructing DLTS plots to directly investigate the shapes of defects and the defect distribution at the p/n junction.

Our findings indicate the validity of this analytical method for imaging of defect depth profile.

## Additional Information

**How to cite this article**: Heo, S. *et al*. Defect visualization of Cu(InGa)(SeS)_2_ thin films using DLTS measurement. *Sci. Rep*. **6**, 30554; doi: 10.1038/srep30554 (2016).

## Supplementary Material

Supplementary Information

## Figures and Tables

**Table 1:** Summary of the CIGSS samples investigated in this study.

Sample	I–V Measurement				Eg from EQE (eV)	Voc Loss	Capacitance Measurement			
	Jsc	Voc	FF	Eff. (%)			Carrier density from CV (cm^−3^)	SCR width from CV (nm)	Free carrier from DLCP (cm^−3^)	RT density from DLCP (cm^−3^)
PJ	37.0	0.650	66.9	16.09	1.181	0.537	2.1 × 10^16^	250	3.4 × 10^15^	2.45 × 10^15^
PK	37.03	0.673	66.8	16.62	1.13	0.457	7.0 × 10^15^	480	1.5 × 10^15^	5.40 × 10^15^

**Table 2:** Band gap profile and valence band parameters calculated from the Auger profile and XPS results.

Sample	Band gap profile				VBM from XPS	
	E_g1_(eV)	E_g2_(eV)	E_g min_(eV)	E_gmin position_(μm)	Top surface (eV)	Center (eV)
PJ	1.3	1.55	1.105	0.3	−0.62	−0.58
PK	1.26	1.52	1.085	0.53	−0.42	−0.57

**Table 3:** Defect parameters (activation energy, density, and cross section) calculated using DLTS.

	PJ sample				PK sample		
	H1	H2	E1	E2	H1	H2	E1
Et (eV)	0.13	0.29	0.18	0.47	0.11	0.35	0.23
Nt (10^14 ^cm^−3^)	3.1	5.1	0.8	8.1	0.8	0.08	1.2
Cross section (σ, cm^2^)	1.2 × 10^−16^	7.0 × 10^−17^	1.3 × 10^−16^	6.8 × 10^−17^	1.3 × 10^−16^	7.4 × 10^−17^	9.5 × 10^−17^
