# Supplementary material for: Defect visualization of Cu(InGa)(SeS)2 thin films using DLTS measurement
Source: Sci Rep. 2016 Aug 1;6:30554. doi: 10.1038/srep30554 (PMC4967860; doi:10.1038/srep30554)
Supplement: Supplementary Information [file srep30554-s1.pdf]

Supplement Information

Defect visualization of $Cu(InGa)(SeS)_2$ thin films using DLTS measurement

Sung Heo[1,5], JaeGwan Chung[1], Hyung-Ik Lee[1], Junho Lee[1], Jong-Bong Park[1], Eunae Cho[1], KiHong Kim[1], Seong Heon Kim[1], Gyeong Su Park[1], Dongho Lee[2*], Jaehan Lee[2], Junggyu Nam[2], JungYup Yang[2], Dongwha Lee [3], Hoon Young Cho [3], Hee Jae Kang[4], Pyung-Ho Choi[5], and Byoung-Deog Choi [5**]

[1] *Analytical Engineering Group, Samsung Advanced Institute of Technology, 130, Samsung-ro, Yeongtong-gu, Suwon-si, Gyeonggi-do, Korea, 443-803*

[2] *PV Development Team, Energy Solution Business Division, Samsung SDI, 467, Beonyeong-ro, Cheonan-si, Chungcheongnam-do, Korea 331-330*

[3] *Department of Physics, Dongguk University, 100-715, Korea*

[4] *Department of Physics, Chungbuk National University, Cheongju, 28644, Korea*

[5] *College of Information and Communication Engineering, Sungkyunkwan University, Cheoncheon-dong 300, Jangan-gu, Suwon 440-746, Korea*

* E-mail: dhlee0333@gmail.com

**E-mail: bdchoi@skku.edu

Measurement methods and conditions

The solar cell performance was characterized under standard conditions: AM 1.5G irradiation at 25℃. For the temperature-dependent electrical measurements, we utilized a liquid nitrogen (LN2) cryostat with a temperature range of 80 to 300 K. The sample temperature was determined by measuring the cold finger temperature with a calibrated Si diode attached to the back of the glass substrate. The temperature was controlled with a Lake Shore Cryotronics 335 temperature controller. The current-voltage-temperature (IVT), capacitance-voltage (CV), and drive-level capacitance profiling (DLCP) measurements were performed with an Agilent Technologies B1500A semiconductor device analyzer. Room temperature carrier density and free carrier density were measured using DLCP at 300 K and 100 K and a 1 kHz frequency, respectively. The AC amplitude of DLCP was varied from 40 mV to 400 mV. The optical response characteristics of the solar cells were measured with an external quantum efficiency (EQE) system with spectral sensitivity (CEP-25BX, BUNKOH-KEIKI), which was calibrated with a Si reference cell of VLSI standard. The EQE was measured from 300 nm to 1300 nm in 10 nm steps.

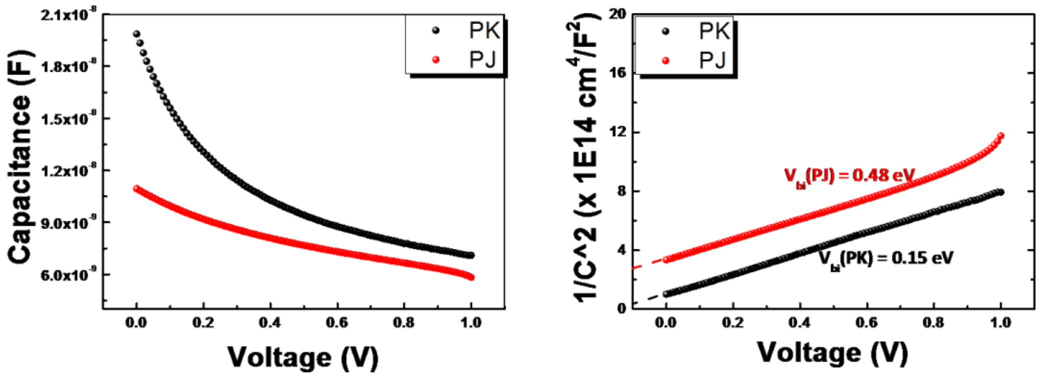

Supplementary Figure 1. C-V curve and C-V characteristics of B-doped ZnO/CIGSS samples with applied voltages.

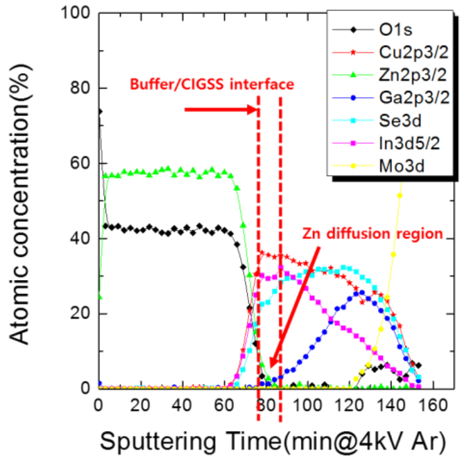

Supplementary Figure 2. The XPS depth profile of the PJ sample. Zn diffusion into the CIGS thin film is confirmed and indicates that a homo pn junction is formed.

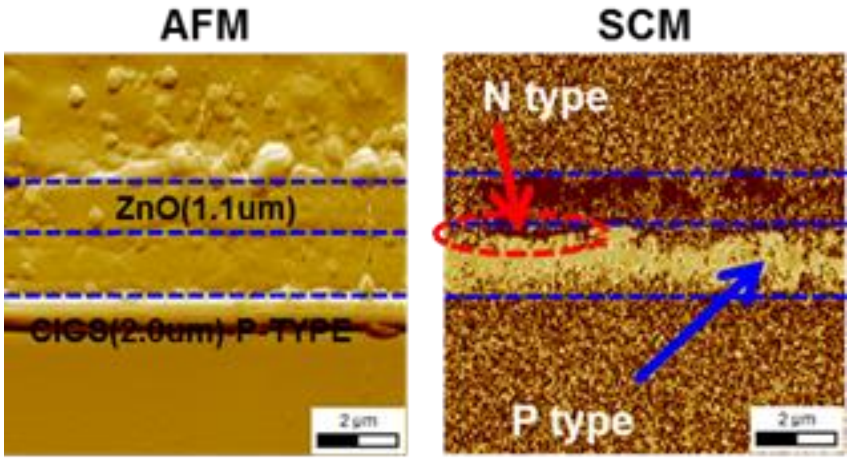

Supplementary Figure 3. The AFM and SCM images of the PJ sample. The contrast in the SCM image is the difference in CIGSS area. The bright area of contrast is p type, and the dark areas represent the n type. This result indicates that a homo-junction is formed inside the CIGSS film.
